# Long-term observations from Antarctica demonstrate that mismatched scales of fisheries management and predator-prey interaction lead to erroneous conclusions about precaution

**DOI:** 10.1038/s41598-020-59223-9

**Published:** 2020-02-11

**Authors:** George M. Watters, Jefferson T. Hinke, Christian S. Reiss

**Affiliations:** Antarctic Ecosystem Research Division, Southwest Fisheries Science Center, National Marine Fisheries Service, National Oceanic and Atmospheric Administration, La Jolla, California 92037 USA

**Keywords:** Conservation biology, Ecosystem ecology

## Abstract

Low catch limits for forage species are often considered to be precautionary measures that can help conserve marine predators. Difficulties measuring the impacts of fisheries removals on dependent predators maintain this perspective, but consideration of the spatio-temporal scales over which forage species, their predators, and fisheries interact can aid assessment of whether low catch limits are as precautionary as presumed. Antarctic krill are targeted by the largest fishery in the Southern Ocean and are key forage for numerous predators. Current krill removals are considered precautionary and have not been previously observed to affect krill-dependent predators, like penguins. Using a hierarchical model and 30+ years of monitoring data, we show that expected penguin performance was reduced when local harvest rates of krill were ≥0.1, and this effect was similar in magnitude to that of poor environmental conditions. With continued climate warming and high local harvest rates, future observations of penguin performance are predicted to be below the long-term mean with a probability of 0.77. Catch limits that are considered precautionary for forage species simply because the limit is a small proportion of the species’ standing biomass may not be precautionary for their predators.

## Introduction

To conserve large fishes, seabirds, and marine mammals, many stakeholders advocate precautionary management of fisheries that target forage species (e.g., krill, anchovies, and sardines). One strategy to conserve predators is to reserve some proportion of their prey^1^, perhaps by establishing a low catch limit for the fisheries that target the forage populations or stocks^2^. However, fishing activities may concentrate where target species are profitably caught, potentially increasing local harvest rates above intended levels^3^. If management fails to prevent concentrated fishing where dependent predators forage, these predators may be impacted despite a low overall catch limit. From an ecosystem perspective, the level of precaution implied by a low catch limit may be better-assessed relative to the time and space scales over which forage species, their predators, and fisheries interact.

Assessing whether catch limits are precautionary from an ecosystem perspective is challenging because the impacts of forage-fish fisheries on predators are difficult to measure^4–6^. This difficulty arises because predators respond to many drivers, including environmental conditions and food-web interactions that are modulated by competition and responses to the availability of alternative prey. Reducing uncertainty to draw unambiguous inference about fisheries impacts on predators requires data that disentangle the effects of fishing from those of the environment and match the temporal and spatial scales of predator life histories, predator-prey interactions, and fishery catches. Data of this nature may not be available at the broad scale of the forage stock, but may be so on smaller scales. Experimental approaches to estimate the effects of fishing are possible^7^, but such experiments are rare and can be controversial^8^. An alternative to experimentation is to leverage long-term observational data that capture natural and anthropogenic variations in the focal system. Such long-term studies in locations where the foraging ranges of predators overlap in time and space with locally intense fishing operations are also rare, but the observations provided by these studies may include contrasts that are sufficient to improve inference about the impacts of forage-fish fisheries on dependent predators.

The Antarctic marine ecosystem provides a useful case study for assessing whether a catch limit established at a regional scale is precautionary given locally high harvest rates. Around the Antarctic Peninsula (AP), Antarctic krill (*Euphausia superba*) are the target of the largest fishery in the Southern Ocean^9^ and a key forage species for fishes, seabirds, and marine mammals^10^. The current catch limit for krill in the entire southwest Atlantic sector of the Southern Ocean is 620,000 tons^3^. This catch limit is spatially divided across four statistical subareas (48.1, 48.2, 48.3 and 48.4; defined by the Commission for the Conservation of Antarctic Marine Living Resources) to reduce the risks of negative impacts on krill-dependent predators in the region. The catch limit in Subarea 48.1, which surrounds the AP, is 155,000 tons, representing <1% of the estimated standing stock of krill (60.3 Mt) in the four subareas^3^. The catch limit in Subarea 48.1 is achieved regularly, and catches are more concentrated in space and time than ever before^9^. Adélie (*Pygoscelis adeliae*), chinstrap (*P. antarcticus*), and gentoo (*P. papua*) penguins that breed around the AP feed on a mixed assemblage of krill, fish, and other invertebrates^11^, but krill are the predominant prey of these seabirds^12,13^. In recent decades, the standing biomass of krill near the AP has varied by two orders of magnitude due to variations in the physical and biological environment^14–17^. The production of penguin populations around the AP is assumed to be linked to the availability of krill^18^, but previous attempts to relate penguin performance with changes in krill biomass have either been unsuccessful^18,19^ or based on broad generalizations inferred from trends in penguin recruitment and abundance^20,21^. The low regional catch limit for krill and the lack of a quantitative relationship between krill biomass and penguin performance around the AP have been used to support arguments that current management of the krill fishery is precautionary^3^. We suggest, however, that the concentration of krill catches, in time and space, acts to locally increase the vulnerability of penguins to the indirect impacts of fishing despite the low regional catch limit.

**Figure 1:**
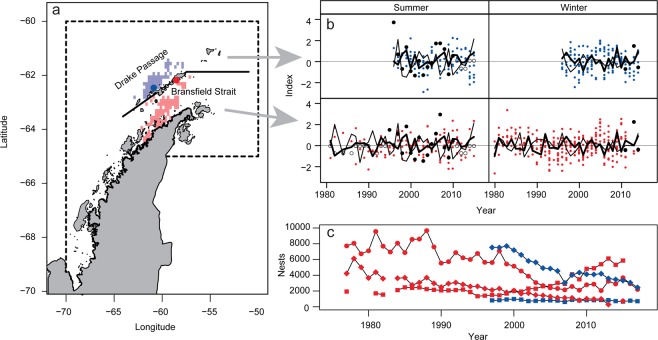
Study area, standardized indices, and trends in penguin abundance. (**a**) Study area, noting Cape Shirreff (blue dot), Copacabana (red dot), and Subarea 48.1 (dashed line); the thick black line separates the Bransfield Strait and Drake Passage strata, and, within these strata, areas of penguin and fishery overlap (adapted from^26^ and made available under the Creative Commons CC0 public domain dedication) are respectively colored light red and light blue. (**b**) Standardized indices of penguin performance during summer and winter at Cape Shirreff (blue) and Copacabana (red), acoustic survey (solid black circles) and imputed (open black circles) estimates of local krill biomass, and the Oceanic Niño Index (thin black line) and Southern Annular Mode (thick black line). (**c**) Population trends for Adélie (circles), chinstrap (diamonds) and gentoo (squares) penguins (colors as in **a**) (data prior to 2005 replotted from^20^). Adapted with permission from Springer Nature: Springer-Verlag *Oecologia* Divergent responses of *Pygoscelis* penguins reveal a common environmental driver, J.T. Hinke, K. Salwicka, S.G. Trivelpiece, G.M. Watters, and W.Z. Trivelpiece, © Springer-Verlag 2007.

We investigated the effects of krill fishing on penguins near the AP consistent with best-practices^5^. Briefly, we compiled time-series data on 20 indices of penguin performance (e.g., foraging-trip duration, post-hatch breeding success, relative cohort strength, fledging mass) at two field sites in the South Shetland Islands^20^ and on krill biomass^22^ in the Bransfield Strait and the northern strata of an established survey grid^23^ (hereafter the Drake Passage stratum). We used the Oceanic Niño Index (ONI) and the Southern Annular Mode (SAM) as proxy indices of environmental conditions that respectively affect penguins^24^ and krill^25^. We used recent tracking data^26^ to match, in time and space, the penguin-performance indices with the estimates of local (stratum-specific) krill biomass and local harvest rates (stratum-specific krill catch divided by stratum-specific krill biomass). We fitted a hierarchical Bayesian model to the integrated data set, first imputing missing estimates of local krill biomass based on its relationship to the sign of the SAM during summer and then estimating the effects of the ONI, local krill biomass, and local harvest rate on penguin performance. Our integrated data characterize a highly variable ecosystem within which penguin performance has responded to fishing while some of our study populations have declined as others have increased (Fig. 1, with panels a and c respectively adapted from^26^ and including data from^20^; see Methods for further detail).

## Results and Discussion

**Figure 2:**
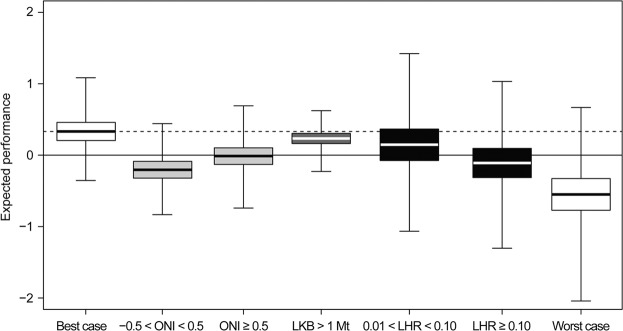
Expected performance of penguins attributable to the marginal effects of the Oceanic Niño Index (ONI), local krill biomass (LKB) and local harvest rate (LHR) relative to the “best case**”**. The best case is ONI ≤ −0.5 °C; LKB ≤ 1 Mt; and LHR ≤ 0.01. The “worst case” is −0.5 °C < ONI < 0.5 °C; LKB > 1 Mt; and LHR ≥ 0.1. The median, interquartile range, and range of the posterior expectations are indicated by each boxplot. Reference lines respectively indicate the median expected performance in the best case (dashed line) and the long-term mean performance.

Variations in local krill biomass (LKB), environmental conditions, and local harvest rate (LHR) correlated with penguin performance, but variation in LKB alone had the smallest effect. To aid interpretation of our results, we defined the “best case” as conditions with ONI ≤ −0.5 °C, LKB ≤ 1 Mt, and LHR* ≤ *0.01. Although it seems counterintuitive that the best case includes low LKB, some indices of penguin performance decrease when penguins forage on small krill^20^, and krill biomass is generally greatest when large cohorts of small krill recruit to the adult population^16^. Relative to the best case, a marginal increase in LKB (to a level >1 Mt) had the smallest effect on penguin performance (Fig. 2) and had the lowest probability (0.7) that expected performance was reduced from the best case (Table 1). The probability that a marginal increase in LKB reduced expected penguin performance below the long-term mean performance was ≤0.04 (Table 1). The insensitivity of penguin performance to variation in LKB corroborates previous failures to parameterize a functional response^3,18,19^ and seems consistent with view that krill biomass in the AP is generally sufficient to support penguin production^27^.

**Table 1:** Posterior and posterior predictive probabilities that expected performance given the effects indicated in the left-most column are less than the expected performance given effects or conditions indicated in the column headings.

	Best case	−0.5 °C < ONI < 0.5 °C	ONI ≥ 0.5 °C	Long-term mean
Best case				0.04 (0.37)
−0.5 °C < ONI < 0.5 °C	1.00			0.88 (0.58)
ONI ≥ 0.5 °C	0.99			0.53 (0.50)
LKB > 1 Mt	0.70	0.02	0.13	0.01 (0.41)
0.01 < LHR < 0.10	0.75	0.17	0.31	0.33 (0.44)
LHR ≥ 0.1	0.93	0.40	0.61	0.64 (0.54)
Worst case	0.99			0.99 (0.77)

Posterior predictive probabilities are indicated in parentheses. ONI is the Oceanic Niño Index, LKB is local krill biomass, and LHR is local harvest rate. In the “best case” ONI ≤ −0.5 °C; LKB ≤ 1 Mt; and LHR ≤ 0.01. In the “worst case” −0.5 °C < ONI < 0.5 °C; LKB > 1 Mt; and LHR ≥ 0.1.

Warm temperatures (ONI > −0.5 °C) and high LHR (≥0.1) decreased penguin performance, and the effects of these two factors were similar (Fig. 2). The probabilities that the marginal effects of intermediate ONI (−0.5 °C < ONI < 0.5 °C) and high LHR caused expected performance to be less than that of the best case were ≥0.93 (Table 1). The probabilities that these marginal effects caused expected penguin performance to be less than the long-term mean were ≥0.64 (Table 1). In contrast to the best case, we defined the “worst case” as conditions with intermediate ONI, high LKB, and high LHR. Relative to the best case, the worst case caused expected penguin performance to be below the long-term mean with a probability of 0.99 (Table 1). We have little doubt that our study populations responded to both the environment and fishing.

**Figure 3:**
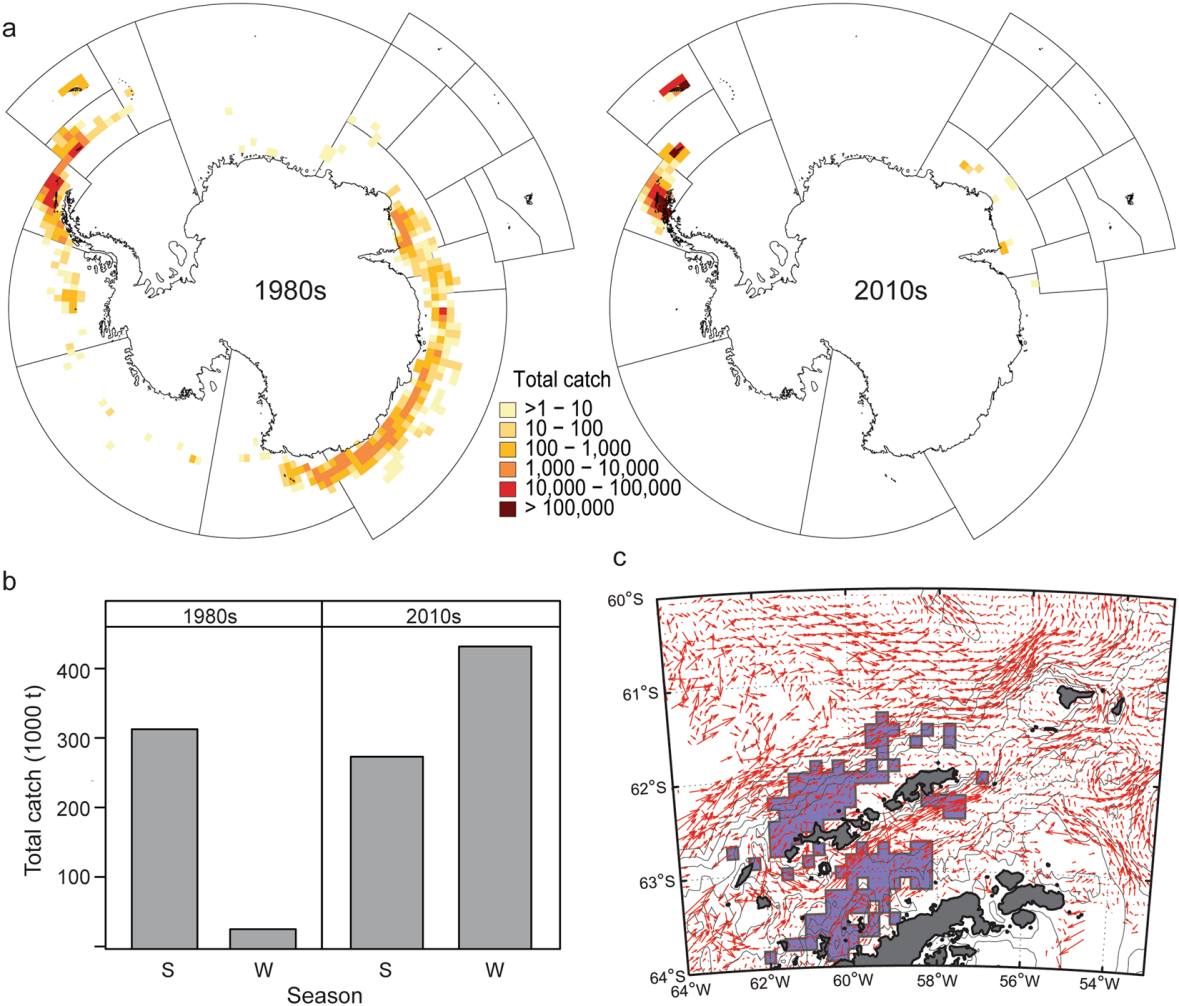
Mechanisms by which krill fishing has impacted penguins. (**a**) Catches (t) contracted from a circumpolar distribution of low catches in the 1980s to higher catches concentrated in the southwest Atlantic sector of the Southern Ocean since 2010. Adapted, with permission from the Secretariat of the Commission for the Conservation of Antarctic Marine Living Resources, from^29^. (**b**) Catches, by decade, taken from Statistical Subarea 48.1 (see Fig. 1) during summer (S) and winter (W). (**c**) Area of overlap (adapted from^26^ and made available under the Creative Commons CC0 public domain dedication) between foraging predators and the fishery krill fishery (shaded polygons) with surface water movements from drifters (red arrows). Large arrows indicate stronger flows.

Recent increases in the spatial and temporal concentration of krill catches^9,28^ provide a plausible mechanism by which the fishery has affected penguins. During the development of the krill fishery, catches contracted from a circumpolar distribution to local hotspots near the AP, the South Orkney Islands, and South Georgia (Fig. 3a, adapted from^29^). In our Bransfield Strait and Drake Passage strata, annual catches taken prior to 2010 averaged about 34,000 t/yr^30^, and LHRs ranged from 0 to 0.01. From 2010 through 2016, catches averaged about 121,000 t/yr^30^, and season-specific LHRs ranged from 0 to 0.32, with rates ≥0.1 occurring 20% of the time in the Bransfield Strait and 10% of the time in the Drake Passage (Supplementary Data S12). For reference, the target harvest rate used to establish precautionary catch limits for the Antarctic krill fishery is 0.093^3^. All instances when the LHR exceeded 0.1 occurred during winter. Winter catches in the AP region have increased over time and now exceed the total catch taken during summer (Fig. 3b).

Increased catches taken at smaller spatial scales during winter have increased the frequency of poor penguin performance and could reduce future recruitment. If the future is represented by our worst case (Fig. 2), the posterior predictive distributions from our model indicate that any performance index, including recruitment, would be less than its long-term mean with a probability of 0.77 (Table 1). An increased probability of poor recruitment raises the risk of population declines, as has been shown for Adélie penguins^31^. Although penguins may compensate so that their performance is not reduced after the birds are “challenged”, e.g.^32^, the population-level consequences of reductions in performance indices other than recruitment (e.g., longer foraging trips, lower adult mass at lay) are not always clear^4,5^. Given the consequences of poor recruitment and the uncertain impacts from other reductions in performance, the fine-scale concentration of catches taken under the umbrella of a regional catch limit established for an entire year does not seem to be as precautionary as presumed.

Climate change has implications for whether management of the krill fishery will be precautionary in the future. It is reasonable to expect that warmer conditions will become more frequent in the AP^33^, increasing the probability of negative impacts on penguins. Even as climate changes in the AP, we expect there will be “good” years in which penguin performance is above average. However, because fishery effects on penguin performance are of similar magnitude to those of poor environmental conditions (Fig. 2), performance during good years might nevertheless be reduced if management of the krill fishery unintentionally facilitates high LHRs. If the current management strategy for krill is maintained over the long term, future years like our worst case scenario seem more likely given the propensity of the krill fishery to target hotspots, the regional scale of the current catch limit, and the expectation of continued warming. As noted previously, such conditions are predicted to cause expected penguin performance to fall below its long-term mean with a probability of 0.77 (Table 1).

The recovery of cetacean populations in the Southern Ocean also has implications for whether management of the krill fishery will be precautionary. In the southwest Atlantic sector of the Southern Ocean, humpback whale populations have increased rapidly in the last 30 years, and recent abundance estimates suggest these populations have recovered to levels approximating their pre-exploitation abundances^34^. On one hand, such recoveries of large cetaceans in the Southern Ocean may reduce the “krill surplus”^35^ that followed industrial whaling. Alternatively, increased krill consumption by whales may fertilize surface waters and increase the productivity of the ecosystem^36,37^. Cetaceans forage in areas where our study penguins overlap with the krill fishery^38^, and the effects of increasing cetacean populations on the structure and function of the ecosystem may affect penguin performance. Determining whether future management of the krill fishery is precautionary will benefit from consideration of such ecosystem perspectives by effectively improving estimates of LKB, and thus LHR.

At least two caveats might bias the LHRs computed here. First, our estimates of LHR might be biased high if the catches were taken from areas in which the throughput of krill was sufficient to replenish local standing stocks. However, penguins foraging from our monitoring sites overlap with the krill fishery in areas where surface currents are relatively weak (Fig. 3c, with panel c adapted from^26^), and habitat models suggest that chinstrap penguins throughout the AP and South Orkney Islands forage in slow moving water^39^. Second, the ongoing recovery of whale populations in the Southern Ocean has the potential to impact penguin populations^21^. If consumption by baleen whales had indeed reduced the standing stocks of krill in areas where our study penguins overlapped with the krill fishery, the LHRs calculated here might be biased low. However, even in this case, the estimated pattern of decreased penguin performance with increased LHR would remain.

Variations in the environment (here indexed by the ONI) and LKB may affect penguin performance in myriad, complex ways. For example, precipitation has been negatively correlated with both chick survival and fledgling mass^40,41^. Also, when the standing stock of krill is large, small krill are proportionally more abundant^22^, and, when small krill are more abundant, chinstrap penguins take longer foraging trips^20^. The literature provides many examples of links between environmental conditions, the characteristics of the prey field, and penguin performance. Our emphasis here has not been to further elucidate or validate such links. Rather we attempt to place the indirect effects of krill fishing in context with these other drivers of penguin performance, which offers a new perspective.

In areas with reduced throughput, fishing may reduce the availably of krill to penguins via absolute reductions in krill biomass (exploitative competition) or from changing the structure of krill swarms or their distribution in the water column (interference competition). Distinguishing these alternatives is difficult in general^5^ and not possible here. However, concentrated krill fishing in hotspots is associated with sequential reductions in catch per unit fishing effort followed by vessel movements to new areas^28^. Furthermore, though high LHRs caused reductions in penguin performance (Fig. 2), these occasions were not coincident with biomasses that were also low enough to reduce penguin performance. In combination, our results thus suggest that interference competition has occurred around the AP, whereby krill fishing was sufficient to change the structure or distribution of krill swarms such that both penguins and the fishery itself were impacted. This hypothesis is testable. In our opinion, it is now feasible, using modern acoustic equipment, to estimate what might have been caught without actually bringing krill on deck. Thus, an experiment could be designed in which an acoustic survey is first conducted to estimate LKB; “normal” fishing activities are then conducted, except with open nets so that krill can escape, until more than 10% of the local biomass might have been caught; and penguin performance is simultaneously monitored. It might be useful to compare results from this treatment to those from a reference area in which fishing was conducted with nets that are closed.

Our results demonstrate that it is generally inadvisable to presume the catch limit for a forage species is precautionary simply because the limit is a small proportion of the species’ standing biomass at a regional scale. The dynamic nature of forage species and the fisheries that target them^1,4,42,43^ means that catch limits applied at coarse spatio-temporal scales can result in unintentionally high LHRs that impact predators. Our integrated, long-term monitoring data demonstrate that this has occurred in the Antarctic krill fishery because the implementation of a presumed precautionary approach did not manage the behavior of the fishery to find and exploit hotspots. When the time and space scales of catch limits are coarser than the scales of predator-prey interactions, it is difficult to achieve ecosystem objectives that include predator conservation^6^. Impacts on predators can likely be mitigated by spatial and temporal allocation schemes that broadly distribute catches to account for predator-prey-fishery interactions^44,45^. Failing this, a precautionary approach to conserve predators foraging in fishing hotspots would be to decrease regional catch limits below the levels that are considered precautionary from a single-species perspective.

## Methods

### Strata

We defined two geographical strata to spatially match season-specific penguin foraging locations with estimates of LKB and LHR. These strata were based on areas where foraging penguins and krill fishing overlap^26^ and on the availability of krill biomass estimates from research-vessel surveys^22,23^. We defined the strata as the Drake Passage, combining the western and Elephant Island survey strata of the U.S. AMLR Program^22^, and the Bransfield Strait (Fig. 1a, adapted from^26^).

### Predictors

We quantified variation in LKB using long-term acoustic survey data^22,23^ from the AP collected following standard protocols^46,47^. We computed the density (g/m^2^) of krill for each nautical mile of survey effort, computed the average density within each stratum, and multiplied those averages by the area of each stratum. Summer surveys were conducted in January and February; winter surveys were conducted in August and September. When more than one survey was conducted in a single season (e.g., January and February of the same year), we averaged the biomass estimates from those surveys. Summer surveys cover the period 1996-2011 (Fig. 1b). Winter surveys are available for 2012, 2014, and 2015 (Fig. 1b).

We computed LHRs by matching catches with temporally and spatially coincident estimates of LKB. Catch data were provided by the Secretariat for the Commission for the Conservation of Antarctic Marine Living Resources. We summed these data by season and stratum and computed LHR as local catch/LKB.

We used two climate indices to characterize seasonal variation in environmental conditions (Fig. 1b). We averaged monthly values of the Southern Annular Mode (SAM^48^) for winter (April-September) and summer (October-March) indices. We also computed seasonal averages of the Oceanic Niño Index (ONI)^49^. We assigned summer averages of the SAM and ONI to the second calendar year of the split year in each austral summer (e.g., the average value for October 2004 - March 2005 was assigned to 2005).

We categorized all four predictors. When the SAM is in a positive (negative) phase, westerly winds are stronger and shifted towards the pole^50^, with a resulting increase (decrease) in air temperature^51^ and precipitation^52^ on the western side of the AP. We thus categorized the SAM according to its sign. The ONI defines La Niña and El Niño events when sea-surface temperature anomalies near the equator are respectively ≤−0.5 °C and ≥0.5 °C. Such events interact with the SAM to affect primary production and krill near the Antarctic Peninsula^15^, so we binned the ONI into three categories (ONI ≤ −0.5 °C; −0.5 °C < ONI < 0.5 °C; and ONI ≥ 0.5 °C) using these two temperature thresholds. Estimates of LKB are imprecise^22^ and may be biased by a variety of factors, including the throughput of krill through our strata^53^. Nevertheless, the surveys distinguish periods of relative krill scarcity and abundance. Acoustic estimates of LKB ranged in magnitude from 10^4^ to 10^7^ t. In contrast, krill catches taken within our strata were reported by fishing vessels, treated as known, and ranged in magnitude from ≤10^3^ to 10^5^ t. Given differences in the uncertainties and magnitudes of local biomasses and catches, we categorized estimates of LKB using a threshold of 1 × 10^6^ t (1 Mt) and of LHR using thresholds of 0.01 and 0.1. The threshold for LKB evenly splits the observed orders of magnitude in krill biomass, and that for LHR provides three categories of fishing that reflect low harvest rates, harvest rates up to that used to set catch limits for krill (0.093), and fishing above that level.

### Response – penguin performance

We used monitoring parameters based on observations collected during 1982-2016 at two field camps in the South Shetland Islands to quantify variations in penguin performance. Unless otherwise noted, methods of data collection have been described previously^20,54^. All methods were performed in accordance with relevant guidelines and regulations, including field methods that were reviewed and approved by the University of California San Diego Institutional Animal Care and Use Committee (ID: S05480) and authorized under U.S. Antarctic Conservation Act permits (ID: ACA 2017-012). Some parameters reflect winter conditions (mean clutch-initiation date, mean female and male masses at lay, mean egg density, and relative cohort strength; Fig. 1b). Other parameters reflect conditions during the summer breeding season (fledgling mass, foraging-trip duration, and post-hatch breeding success; Fig. 1b). In all but one case, these parameters integrate over days to months. Relative cohort strength integrates over a period ≥1 year, but we assumed that most of its variation is attributable to survival during the first few months of independence^20^. We transformed mean clutch-initiation dates and foraging-trip durations so that larger values would indicate better performance. The former parameter was transformed to days prior to 31 December (earlier clutch initiations indicate better performance); the latter was transformed to hours <60 hours (shorter trips indicate better performance). Relative cohort strength (proportion of banded penguins resighted in their breeding colonies) and post-hatch breeding success (proportion of chicks crèched) were logit-transformed. Mean egg density was calculated from the total mass and volume of eggs from 2-egg clutches. Egg data were collected during the first week after clutch completion by weighing and measuring the maximum length and width of each egg from 50 nests per species. Egg volume was estimated from its empirical relationship^55^ to egg length and width. We standardized the penguin performance indices specific to each combination of monitoring parameter (transformed or otherwise), species, and site to have zero mean and unit variance (Fig. 1b).

We matched penguin-performance indices to estimates of LKB and LHR in the Bransfield Strait or Drake Passage strata using results from multi-year tracking studies that identify when and where penguins foraged^26^. We matched the winter and summer performance indices for Adélie and gentoo penguins breeding at Copacabana (Fig. 1a, adapted from^26^) with LKB and LHR in the Bransfield Strait. We matched winter indices for chinstrap penguins breeding at Cape Shirreff (Fig. 1a, adapted from^26^) with LKB and LHR in the Drake Passage, while those for gentoo penguins breeding at the same site were matched with predictors in the Bransfield Strait. After matching, we pooled performance indices within 18 bins defined by all combinations of the categorized predictors.

### Model

We fitted an analysis of variance model with two components. The first component imputes missing estimates of LKB because krill surveys were not conducted every year. We modeled LKB as a function of stratum and the sign of the SAM during summer.

1 $$LK{B}_{ij} \sim \,\log \,-\,{\rm{Normal}}({K}_{ij},{\phi }^{2})$$

${K}_{ij}$ is the expected value of $\mathrm{ln}\,LKB$ in stratum $i$ given sign $j$ of the SAM during summer, and ${\phi }^{2}$ is the variance of $\mathrm{ln}\,LKB$. We truncated this likelihood with a lower limit equal to the catch taken from stratum $i$ (so $LK{B}_{ij}$ would not be less than ${{\rm{catch}}}_{i}$) and an upper limit equal to 100 Mt (so $LK{B}_{ij}$ would be less than twice the estimate of krill biomass used to manage the krill fishery^46^). We used results from the acoustic surveys to specify prior distributions for the imputation model.

2 $$\begin{array}{c}{K}_{ij} \sim U(0.1{\bar{k}}_{ij},10{\bar{k}}_{ij})\\ \phi  \sim U(0.1{s}_{ij},10{s}_{ij})\end{array}$$

${\bar{k}}_{ij}$ is the mean $\mathrm{ln}\,LKB$ computed from survey observations in stratum $i$ given sign $j$ of the SAM, and ${s}_{ij}$ is the standard deviation of these log-biomass estimates. We predicted missing estimates of LKB $(LK{B}_{ij}^{\ast })$ from Eq. (1) using the sign of the SAM for summers when acoustic surveys were not conducted. Missing estimates of LHR, denoted as $LH{R}^{\ast }$, were estimated from $\,LK{B}_{ij}^{\ast }$.

3 $$LH{R}_{ij}^{\ast }={{\rm{catch}}}_{i}/LK{B}_{ij}^{\ast }$$

We did not impute missing values for winter.

In the second component of our model, we treated all indices of penguin performance as exchangeable observations, and modeled performance ($P$) as a function of categorized ONI ($o$), LKB ($b$), and LHR ($h$).

4 $$\begin{array}{c}p \sim N(P,{\sigma }^{2})\\ P=\alpha +{\beta }_{1}{o}_{1}+{\beta }_{2}{o}_{2}+{\beta }_{3}b+{\beta }_{4}{h}_{1}+{\beta }_{5}{h}_{2}\end{array}$$

$P$ is the expected performance, and ${\sigma }^{2}$ is the residual variance in performance. $\alpha$ quantifies mean performance across the set of predictor categories, and the parameters ${\beta }_{1},{\beta }_{2},\,{\beta }_{3},{\beta }_{4},\,{\rm{and}}\,{\beta }_{5}\,$quantify the degree to which each predictor causes expected performance to deviate from this mean. We specified prior distributions that limit inference to the study populations and the predictor bins considered here.

5 $$\begin{array}{c}\alpha  \sim N(0,1.0\times {10}^{4})\\ {\beta }_{1} \sim N(0,1.0\times {10}^{4})\\ {\beta }_{2} \sim N(0,1.0\times {10}^{4})\\ {\beta }_{3} \sim N(0,1.0\times {10}^{4})\\ {\beta }_{4} \sim N(0,1.0\times {10}^{4})\\ {\beta }_{5} \sim N(0,1.0\times {10}^{4})\end{array}$$

We specified a half-Cauchy prior distribution for the standard deviation of penguin performance and a uniform hyperprior for the scale parameter ($\omega$) of the half-Cauchy^56,57^.

6 $$\sigma  \sim {\rm{half}}\,-\,{\rm{Cauchy}}({\omega }^{2})$$

7 $$\omega  \sim U(0,2)$$

We specified a design matrix with sum-to-zero contrasts that carries uncertainties in $LK{B}^{\ast }$ and $LH{R}^{\ast }$ through the model.

8 $$\begin{array}{c}{o}_{1}=\left\{\begin{array}{ll}-1 & {\rm{if}}\,{\rm{ONI}}\le -\,0.5\\ 0 & {\rm{if}}\,{\rm{ONI}}\ge 0.5\\ 1 & {\rm{if}}-\,0.5 < {\rm{ONI}} < 0.5\end{array}\right.\\ {o}_{2}=\left\{\begin{array}{ll}-1 & {\rm{if}}\,{\rm{ONI}}\le -\,0.5\\ 0 & {\rm{if}}-\,0.5 < {\rm{ONI}} < 0.5\\ 1 & {\rm{if}}\,{\rm{ONI}}\ge 0.5\end{array}\right.\\ b=\left\{\begin{array}{ll}-1 & {\rm{if}}\,LKB\,{\rm{or}}\,LK{B}^{\ast }\le 1\,{\rm{Mt}}\\ 1 & {\rm{if}}\,LKB\,{\rm{or}}\,LK{B}^{\ast } > 1\,{\rm{Mt}}\end{array}\right.\\ {h}_{1}=\left\{\begin{array}{ll}-1 & {\rm{if}}\,LHR\,{\rm{or}}\,LH{R}^{\ast }\le 0.01\\ 0 & {\rm{if}}\,LHR\,{\rm{or}}\,LH{R}^{\ast }\ge 0.1\\ 1 & {\rm{if}}\,0.01 < LHR\,{\rm{or}}\,LH{R}^{\ast } < 0.1\end{array}\right.\\ {h}_{2}=\left\{\begin{array}{ll}-1 & {\rm{if}}\,LHR\,{\rm{or}}\,LH{R}^{\ast }\le 0.01\\ 0 & {\rm{if}}\,0.01 < LHR\,{\rm{or}}\,LH{R}^{\ast } < 0.1\\ 1 & {\rm{if}}\,LHR\,{\rm{or}}\,LH{R}^{\ast }\ge 0.1\end{array}\right.\end{array}$$

We used JAGS^58^, via R^59^, to sample from the posterior distributions of the model parameters. After 250,000 adaptive and 500,000 burn-in iterations, we sampled 5,000 points (retaining values from every 25th iteration during a further 125,000 iterations) from the posterior distributions characterized by three Monte Carlo chains initiated at different points in the parameter space. We evaluated our model with a variety of diagnostics. Diagnostics, data files, and the code needed to replicate our analysis are provided as Supplemental Information. Three R packages are needed to run our code: rjags^60^, coda^61^, and ggmcmc^62^.

## Supplementary information

Supplementary information

Supplementary Data S1.

Supplementary Data S2.

Supplementary Data S3.

Supplementary Data S4.

Supplementary Data S5.

Supplementary Data S6.

Supplementary Data S7.

Supplementary Data S8.

Supplementary Data S9.

Supplementary Data S10.

Supplementary Data S11.

Supplementary Data S12.

Supplementary Methods S1.

Supplementary Methods S2.

## Data Availability

All model code and data used to conduct this study are included in Supplementary Information files of this published article.
