# Supplementary material for: Long-term observations from Antarctica demonstrate that mismatched scales of fisheries management and predator-prey interaction lead to erroneous conclusions about precaution
Source: Sci Rep. 2020 Feb 11;10:2314. doi: 10.1038/s41598-020-59223-9 (PMC7012885; doi:10.1038/s41598-020-59223-9)
Supplement: Supplementary file 1 — Supplementary information [file 41598_2020_59223_MOESM1_ESM.pdf]

Supplementary Information for

Long-term observations from Antarctica demonstrate that mismatched scales of fisheries management and predator-prey interaction lead to erroneous conclusions about precaution

George M. Watters[1,*], Jefferson T. Hinke[1] & Christian S. Reiss[1]

[1]Antarctic Ecosystem Research Division, Southwest Fisheries Science Center, National Marine Fisheries Service, National Oceanic and Atmospheric Administration. La Jolla, California 92037, United States of America

[*]George.Watters@noaa.gov

Supplementary Note

Diagnostics

We used a suite of common diagnostics to assess our model. In general, after adaptation and burn in, all chains converged on their target distributions. All trace plots illustrated satisfactory mixing (Supplementary Fig. S1), and all point estimates of potential scale reduction factors[1] for the model parameters equaled 1.0 (Supplementary Fig. S2). The absolute values of Geweke Z-scores[2] for the model parameters were mostly, but not universally, ≤ 1.96 when subsets containing 10% of parameter values from the first half of each thinned chain were compared to those from the last half of the same chain (Supplementary Fig. S3). P-values for Heidelberger-Welch stationarity tests[3] were all < 0.05, but associated half-width tests routinely failed for the effect of 0.01 < LHR < 0.10 (β_4 from equation (4) in the main text), even after additional iterations were run. The latter tests all passed when missing estimates of LKB were not imputed (see below). Within-chain autocorrelation coefficients were low at lags > 0 (Supplementary Fig. S4) and averaged < 0.05 at lag 25. Cross-correlations between parameters in the penguin-performance component of our model ranged from -0.76 (between β_4 and β_5) to 0.35 (between β_3 and β_5) (Supplementary Fig. S5). All other cross-correlations between model parameters ranged from -0.14 (between β_4 and $K_{\text{Drake, -SAM}}$) to 0.06 (between β_5 and $K_{\text{Drake, -SAM}}$). Ultimately, the posterior distributions of the parameters in our model appeared to be proper (Supplementary Fig. S6), and a visual check of posterior predictive distributions specific to the 18 combinations of categorized predictors indicated that the model satisfactorily fitted the available observations of penguin performance (Supplementary Fig. S7).

We conducted four sensitivity tests: two to evaluate whether some of our prior distributions were overly informative, and two that represent alternative models. In the first two cases we specified prior distributions on variance hyperparameters that were nominally less informative than those in our final model. We increased the ranges of the priors for the standard deviation of $\ln LKB$ and the scale parameter for the half-Cauchy distribution of the standard deviation in penguin performance by respectively stipulating $\varphi \sim U(0.01 s_{ij}, 100 s_{ij})$ and $\omega \sim U(0, 5)$. The posterior probabilities that the best case, worst case, and marginal effects

of ONI, LKB, and LHR caused expected performance to be less than its long-term mean were insensitive to these alternative prior distributions (Supplementary Table S1). The same was true when we increased the upper limit of the likelihood for LKB during the summer (Supplementary Table S1). Thus, it does not appear that the prior distributions and krill likelihood in our final model were overly prescriptive; our results were driven by our data. Nevertheless, since a few of the diagnostics related to the effects of LKB and LHR in our final model were equivocal (see proceeding paragraph), we conducted a fourth sensitivity test in which we did not impute missing estimates of LKB during summer. When missing biomass estimates were not imputed, the marginal effects of most predictor categories (-0.5°C < ONI < 0.5°C; LKB > 1 Mt; 0.01 < LHR < 0.1; and LHR ≥ 0.1) were estimated to have more negative effects on penguin performance (Supplementary Table S1). In the fourth sensitivity test, only the effect of ONI ≥ 0.5°C was estimated to improve penguin performance relative to our final model. Thus, the basic patterns estimated in our final model, with increased ONI, LKB, and LHR reducing penguin performance relative to the "best case" and with effects of ONI and LHR being of similar magnitude, were not sensitive to whether missing estimates of LKB were imputed (Supplementary Fig. S8).

Supplementary Fig. S1. Traceplots for model parameters in equations (1) and (4) from the main text. Alpha is the parameter α; beta[1] is β_1 (with other betas similarly identified); K[B,-] is $K_{Bransfield,-SAM}$ (with other log krill biomasses similarly identified using B and D to respectively indicate the Bransfield Strait and Drake's Passage strata, and – and + respectively indicating negative and positive SAMs); phi is φ; and sigma is σ. Different colors identify traces from different Monte Carlo chains.

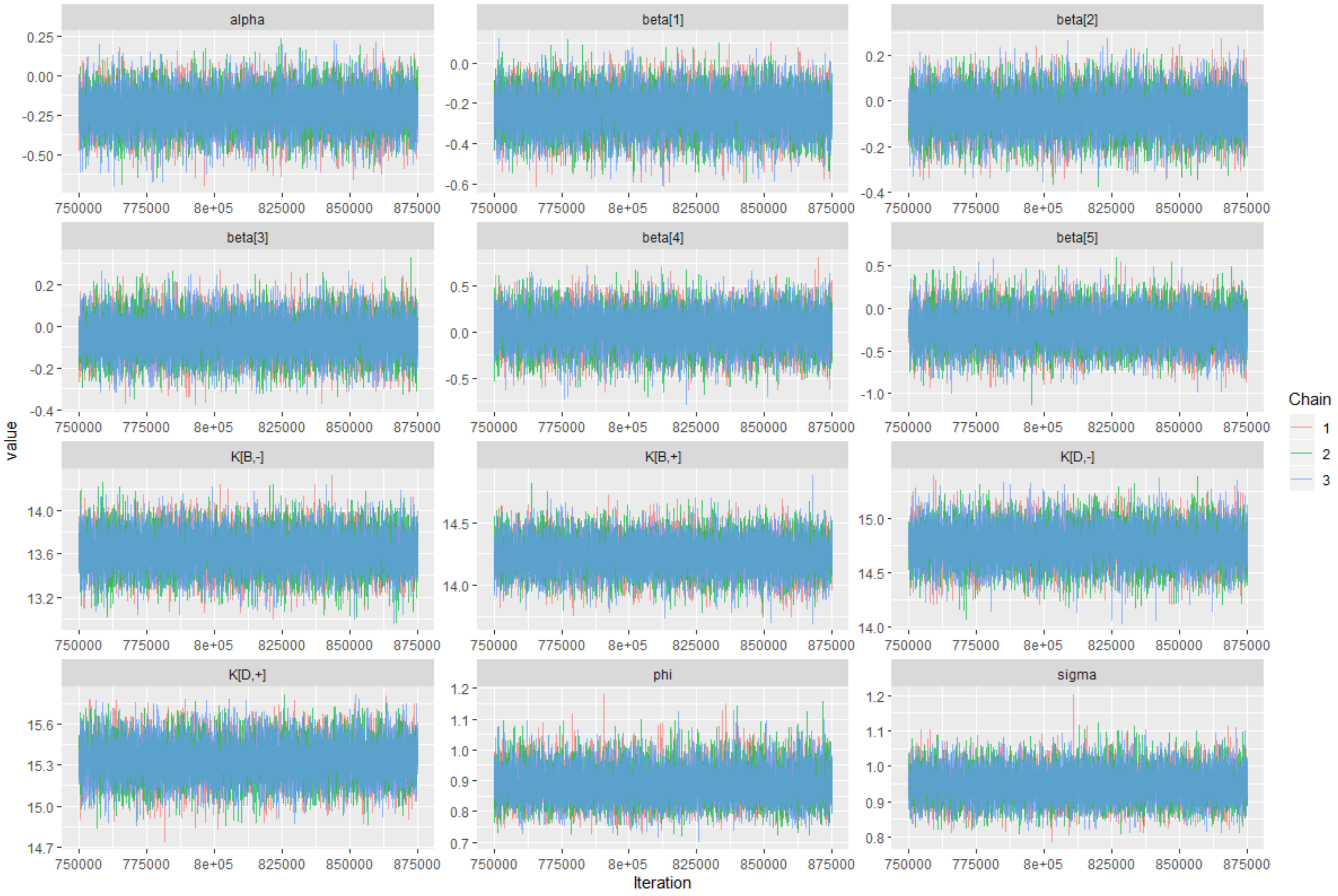

Supplementary Fig. S2. Potential scale reduction factors ($\widehat{R}$) for model parameters in equations (1) and (4) from the main text. Other details as in Supplementary Fig. S1.

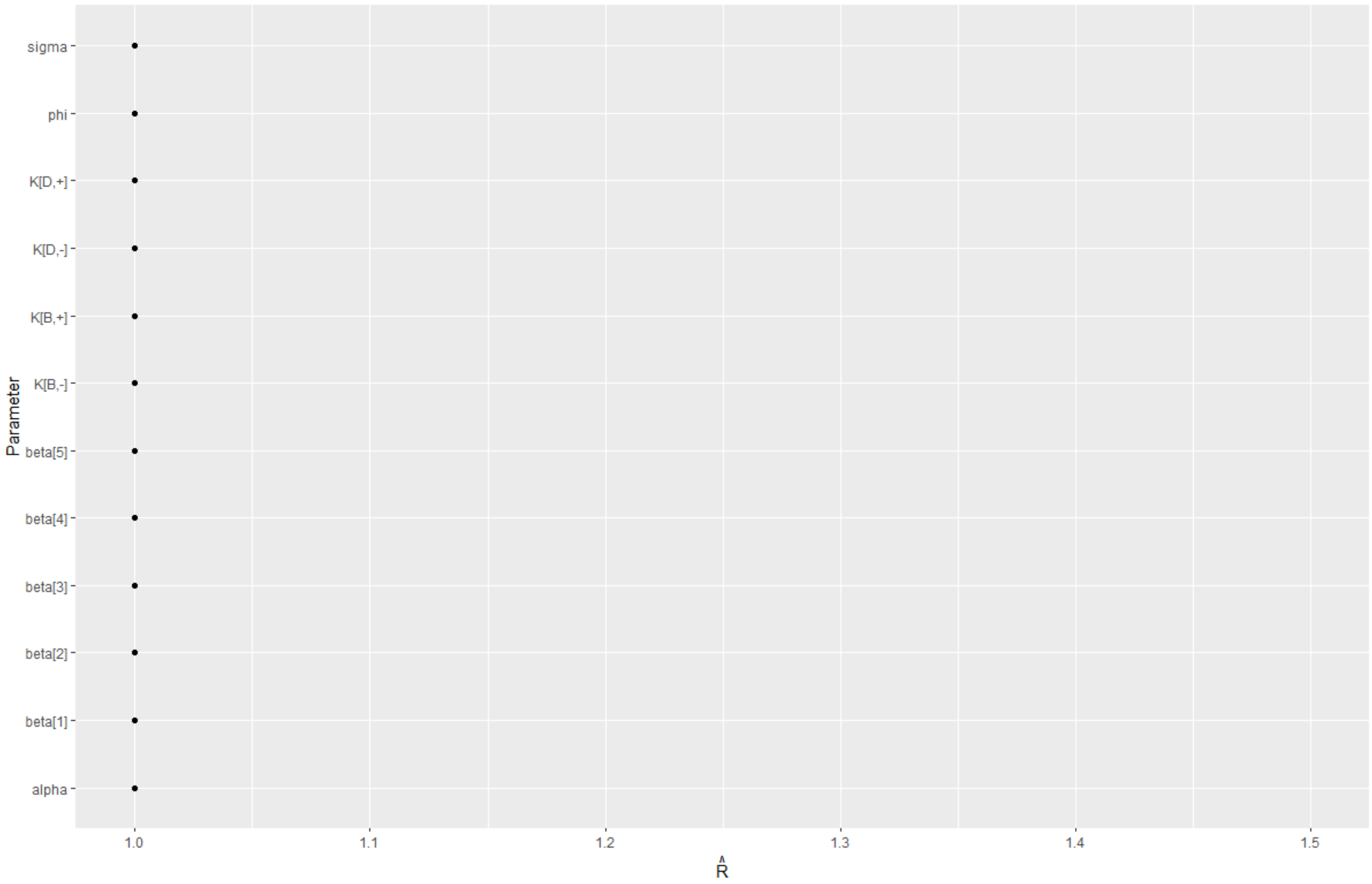

Supplementary Fig. S3. Geweke's Z-scores for model parameters in equations (1) and (4) from the main text. The shaded area identifies scores in the range [-2, 2]. Other details as in Supplementary Fig. S1.

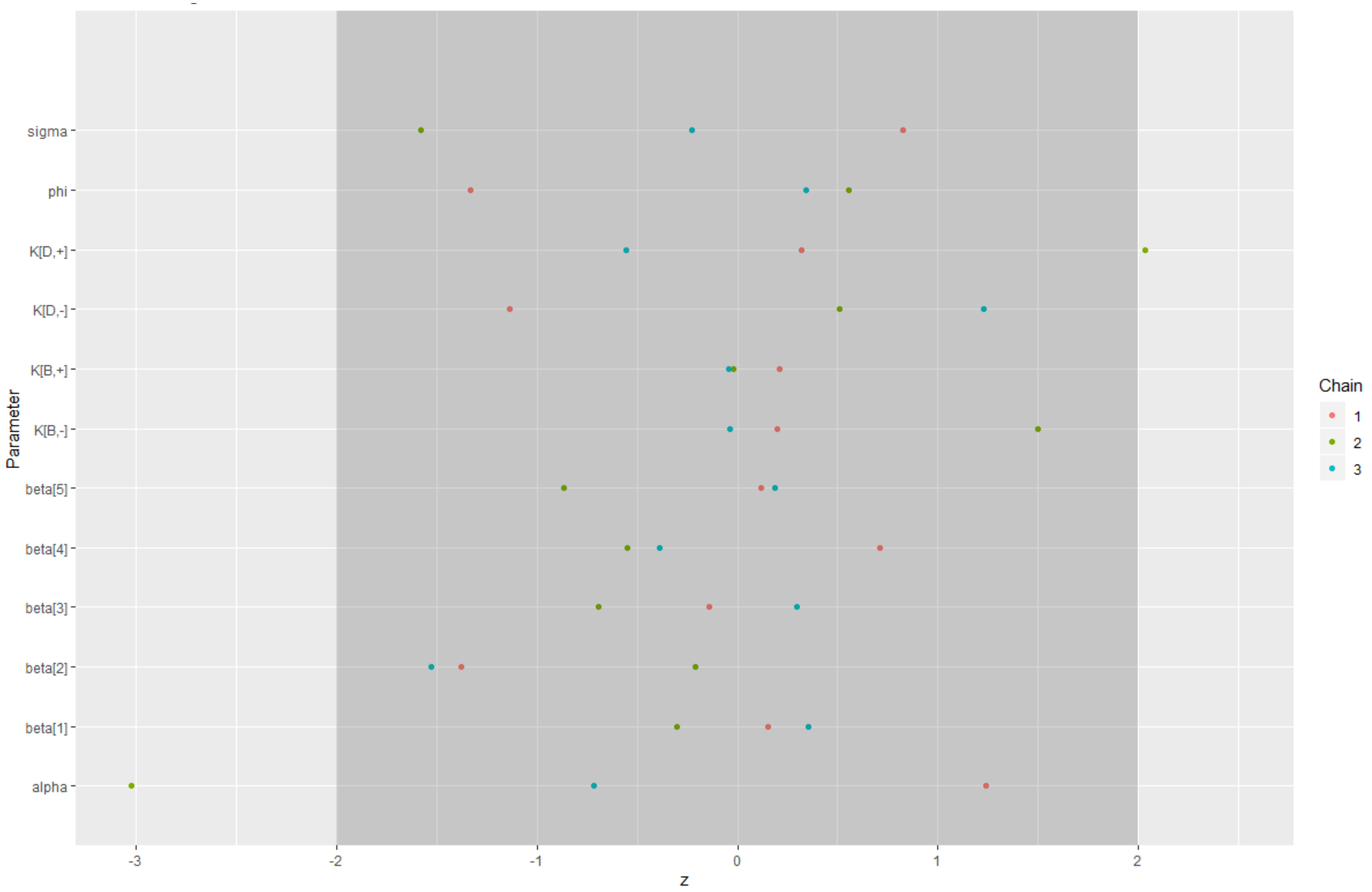

Supplementary Fig. S4. Within-chain autocorrelations of model parameters in equations (1) and (4) from the main text. Other details as in Supplementary Fig. S1.

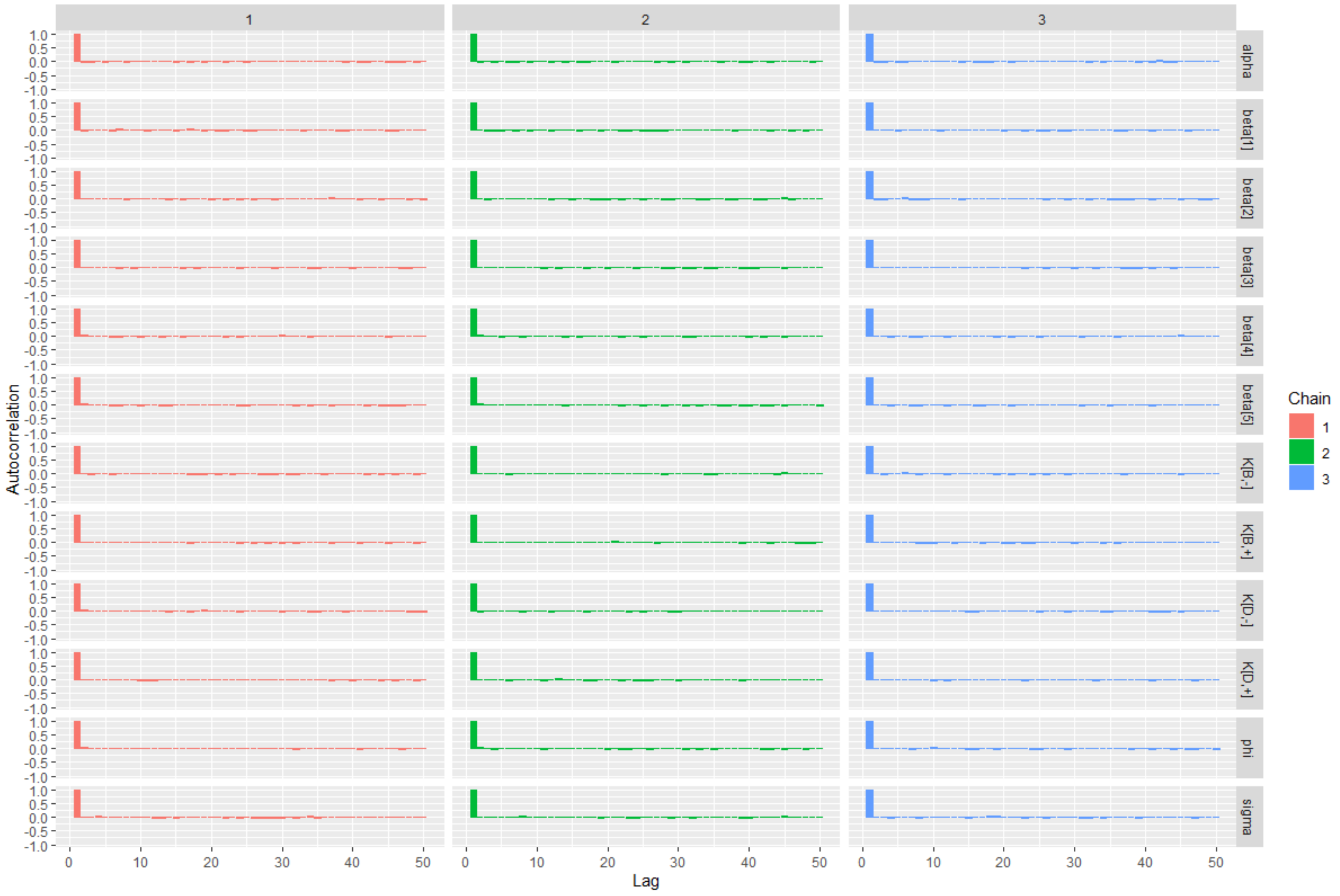

Supplementary Fig. S5. Cross-correlations of model parameters in equations (1) and (4) from the main text. Other details as in Supplementary Fig. S1.

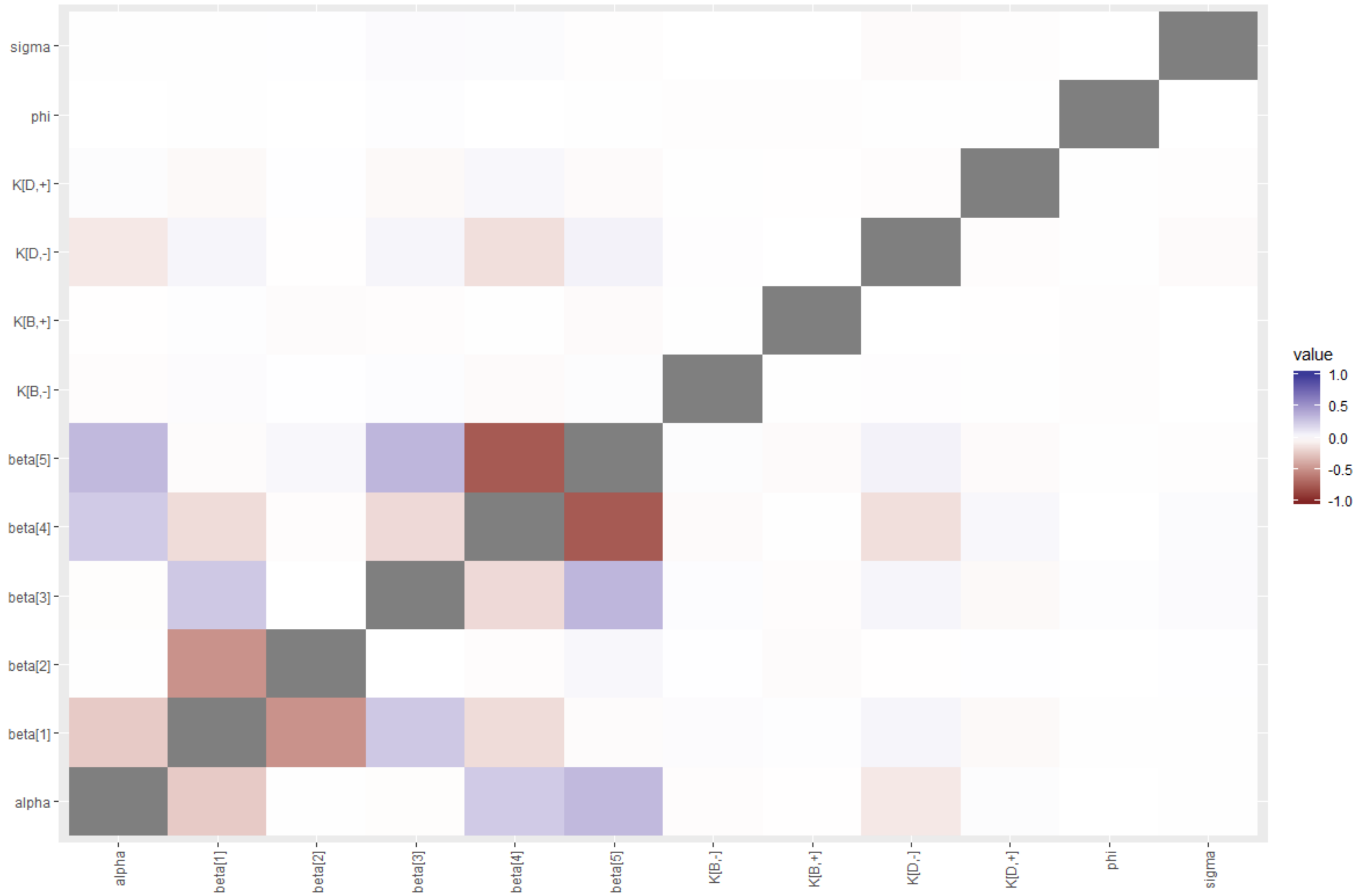

Supplementary Fig. S6. Posterior distributions of model parameters in equations (1) and (4) from the main text. The distributions are composed of parameter values from all three Monte Carlo chains. Other details as in Supplementary Fig. S1.

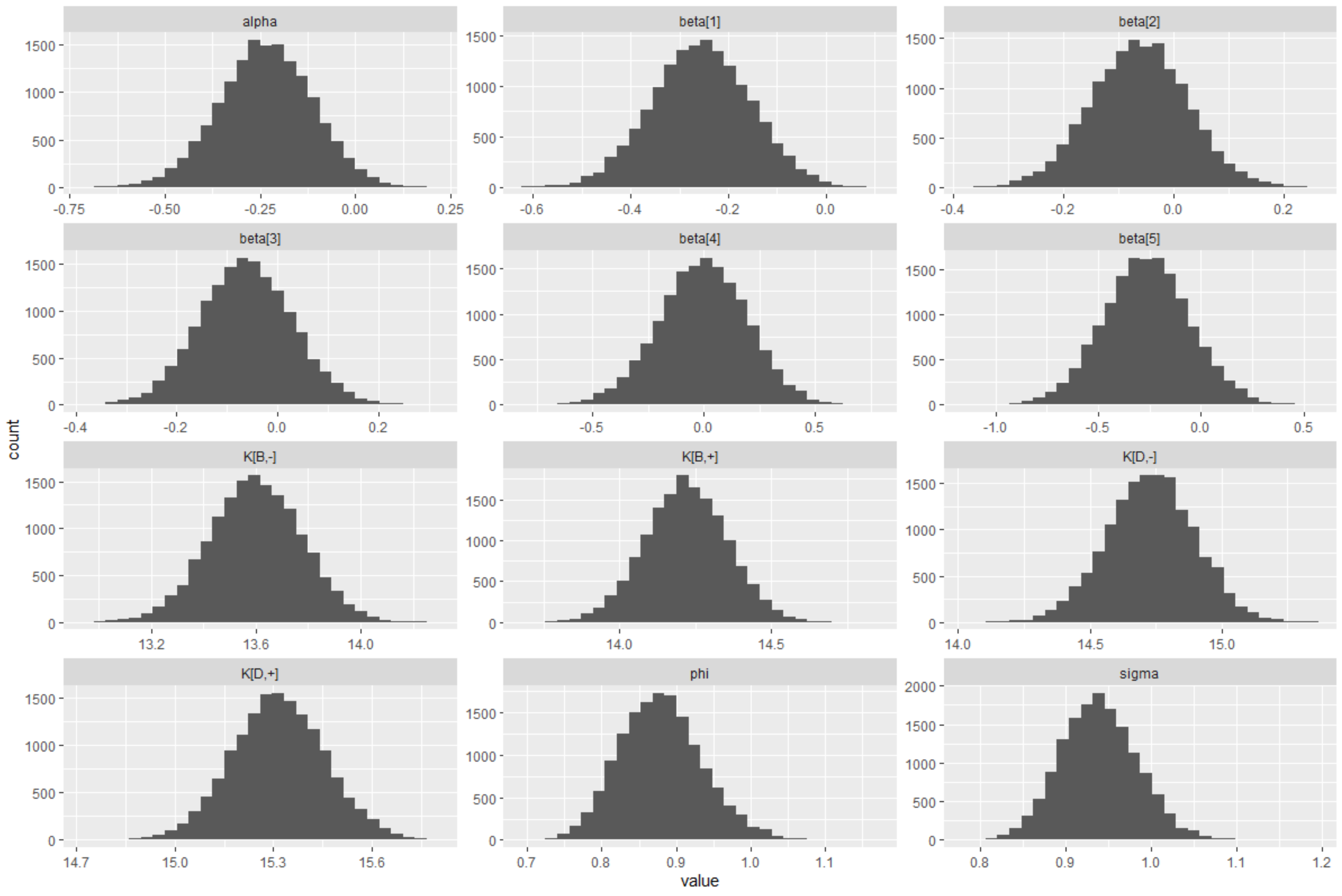

Supplementary Fig. S7. Posterior predictive distributions (boxplots) and jittered observations (colored circles) of penguin performance. Case 1 is ONI ≤ -0.5°C; LKB ≤ 1 Mt; and LHR ≤ 0.01 (the "best case"). Case 2 is ONI ≤ -0.5°C; LKB > 1 Mt; and LHR ≤ 0.01. Case 3 is ONI ≤ -0.5°C; LKB ≤ 1 Mt; and 0.01 < LHR < 0.1. Case 4 is ONI ≤ -0.5°C; LKB > 1 Mt; and 0.01 < LHR < 0.01. Case 5 is ONI ≤ -0.5°C; LKB ≤ 1 Mt; and LHR ≥ 0.1. Case 6 is ONI ≤ -0.5°C; LKB > 1 Mt; and LHR ≥ 0.1. Cases 7-12 are the same as Cases 1-6 except -0.5°C < ONI < 0.5°C; Case 12 is the "worst case." Cases 13-18 are the same as Cases 1-6 except ONI ≥ 0.5°C. Filled red circles represent summer observations when estimates of LKB were available from krill surveys. Open red circles represent summer observations when estimates of LKB were imputed. Blue circles represent winter observations. Boxes characterize interquartile ranges with medians indicated by bold horizontal lines and whiskers extending to 1.5 times these ranges.

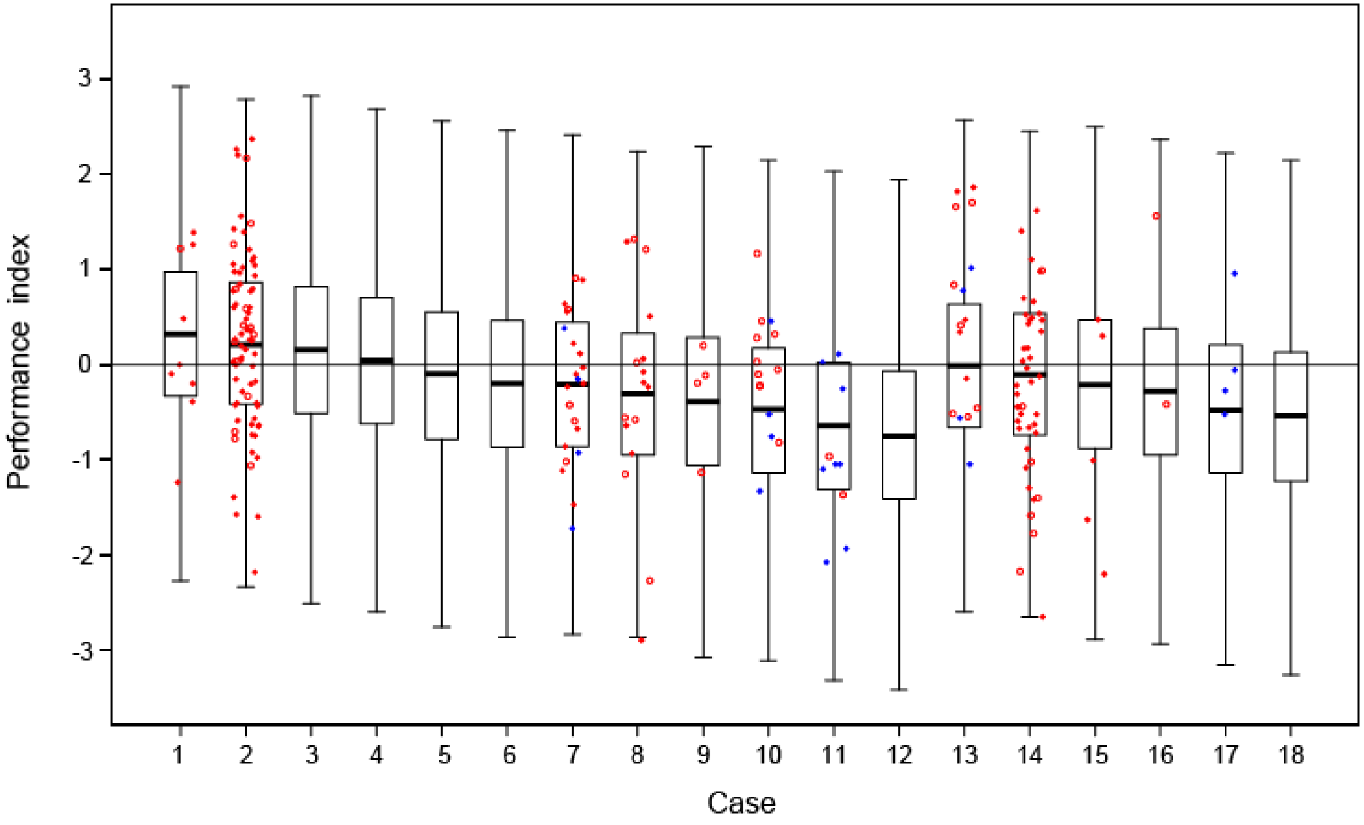

Supplementary Fig. S8. Posterior expectations of penguin performance from the sensitivity test in which missing estimates of LKB were not imputed. The “best case” is ONI ≤ -0.5; LKB ≤ 1 Mt; and LHR ≤ 0.01. The “worst case” is -0.5 < ONI < 0.5; LKB > 1 Mt, and LHR ≥ 0.1. All other cases are marginal deviations from the best case. The median, interquartile range, and range of the posterior expectations are provided for each boxplot. Reference lines respectively indicate the median expected performance in the best case (dashed line) and the long-term mean performance (solid line).

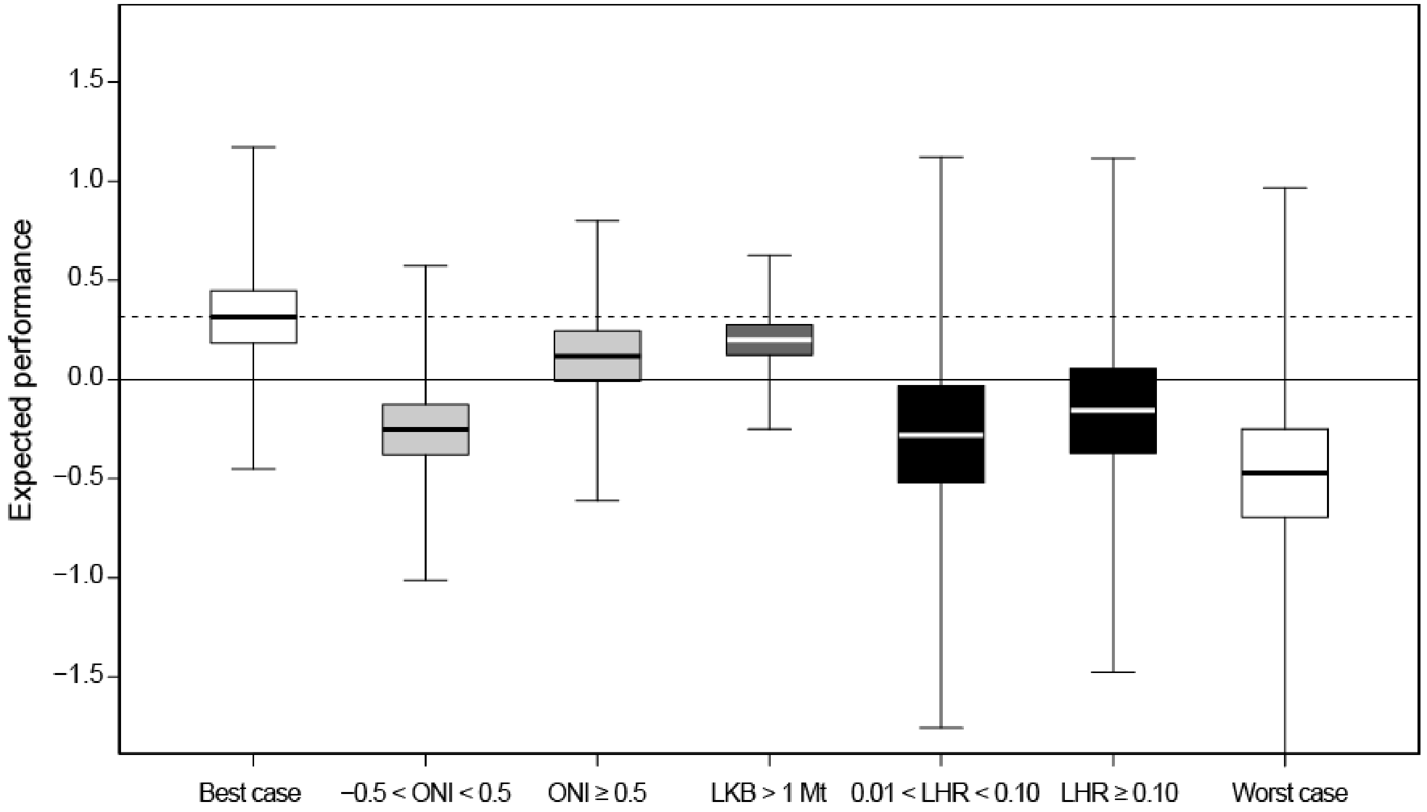

Supplementary Table S1. Posterior probabilities that, given cases indicated in the left-most column, expected penguin performance was less than the expected long-term performance. Values for the final model are from Table 1 in the main text, and values in other columns are from the four sensitivity tests indicated by the column headings. The best case is ONI ≤ -0.5 °C; LKB ≤ 1 Mt; and LHR ≤ 0.01. The worst case is -0.5 °C < ONI < 0.5 °C; LKB > 1 Mt; and LHR ≥ 0.1. All other cases are marginal deviations from the best case.

	Final model	$\varphi \sim U(0.01s_{ij}, 100s_{ij})$	$\omega \sim U(0, 5)$	Upper limit for likelihood of LKB_{ij} = 200 Mt	No imputation
Best case	0.04	0.04	0.04	0.04	0.05
-0.5 °C < ONI < 0.5 °C	0.88	0.88	0.88	0.88	0.91
ONI ≥ 0.5	0.53	0.53	0.53	0.53	0.26
LKB > 1 Mt	0.01	0.02	0.02	0.01	0.04
0.01 < LHR < 0.10	0.33	0.32	0.33	0.33	0.78
LHR ≥ 0.1	0.64	0.64	0.65	0.64	0.69
Worst case	0.99	0.99	0.99	0.99	0.99

Supplementary Data S1 (c1.csv)

Krill catches by year, month, and small scale management unit (SSMU, as defined by the Commission for the Conservation of Antarctic Marine Living Resources).

Supplementary Data S2 (cid.csv)

Mean clutch initiation dates by year, species, and study site.

Supplementary Data S3 (egg.csv)

Egg mass and volume by year, species, and study site.

Supplementary Data S4 (fweight.csv)

Fledgling mass by year, species, and study site.

Supplementary Data S5 (krillsurveywithJoinville.csv)

Estimates of krill biomass from acoustic surveys by year and survey stratum.

Supplementary Data S6 (massatlay.csv)

Adult female and male masses at lay by year, species, and study site.

Supplementary Data S7 (oni.csv)

Values of the Oceanic Niño Index by year and quarter.

Supplementary Data S8 (recruitment.csv)

Relative cohort strength by year, species, and study site.

Supplementary Data S9 (sam.csv)

Values of the Southern Annular Mode by year and month.

Supplementary Data S10 (success.csv)

Data to compute post-hatch breeding success (chicks crèched per chicks hatched) by year, species, and study site.

Supplementary Data S11 (tripduration.csv)

Foraging-trip durations by year, species, and study site.

Supplementary Data S12 (hr.csv)

Krill catches and local harvest rates, including imputed rates, by calendar year, season, and stratum.

Supplementary Methods S1 (localhr.R)

Code to compile, standardize, and match the data sets and then to run the final model.

Supplementary Methods S2 (localhr – no impute.R)

Code to compile, standardize, and match the data sets and then to run a model in which missing estimates of krill biomass during summer are not imputed.

References

1. Gelman, A. & Rubin, D. B. Inference from iterative simulation using multiple sequences. *Statistical Science* **7**, 457-511 (1992).
2. Geweke, J. in *Bayesian Statistics 4* (eds. Bernado, J.M., Berger J.O., Dawid A.P., & Smith A.F.M.) 641-649 (Clarendon Press, Oxford, UK, 1992).
3. Heidelberger, P. & Welch P. D. Simulation run length control in the presence of an initial transient. *Opns Res* **31**, 1109-1144 (1983).
